# Nitrogen Limitation Alters the Response of Specific Genes to Biotic Stress

**DOI:** 10.3390/ijms19113364

**Published:** 2018-10-27

**Authors:** Mahsa Farjad, Martine Rigault, Stéphanie Pateyron, Marie-Laure Martin-Magniette, Anne Krapp, Christian Meyer, Mathilde Fagard

**Affiliations:** 1 Institut Jean-Pierre Bourgin, INRA, AgroParisTech, CNRS, Université Paris-Saclay, 78000 Versailles, France; farjad_mahsa@yahoo.fr (M.F.); martine.rigault@inra.fr (M.R.); anne.krapp@inra.fr (A.K.); christian.meyer@inra.fr (C.M.); 2 Institute of Plant Sciences Paris-Saclay (IPS2), CNRS, INRA, Université Paris-Sud, Université d’Evry, Université Paris-Saclay, Bâtiment 630, Plateau de Moulon, 91192 Gif sur Yvette, France; stephanie.pateyron@inra.fr (S.P.); marie_laure.martin-magniette@agroparistech.fr (M.-L.M.-M.); 3 Institute of Plant Sciences Paris-Saclay (IPS2), CNRS, INRA, Université Paris-Diderot, Sorbonne Paris-Cité, Bâtiment 630, Plateau de Moulon, 91192 Gif sur Yvette, France; 4 UMR MIA-Paris, AgroParisTech, INRA, Université Paris-Saclay, 75005 Paris, France

**Keywords:** multistress, bacterial phytopathogen, nitrogen limitation, Arabidopsis, transcriptome, defense

## Abstract

In their natural environment, plants are generally confronted with multiple co-occurring stresses. However, the interaction between stresses is not well known and transcriptomic data in response to combined stresses remain scarce. This study aims at characterizing the interaction between transcriptomic responses to biotic stress and nitrogen (N) limitation. Plants were grown in low or full N, infected or not with *Erwinia amylovora* (*Ea*) and plant gene expression was analyzed through microarray and qRT-PCR. Most *Ea*-responsive genes had the same profile (induced/repressed) in response to *Ea* in low and full N. In response to stress combination, one third of modulated transcripts responded in a manner that could not be deduced from their response to each individual stress. Many defense-related genes showed a prioritization of their response to biotic stress over their response to N limitation, which was also observed using *Pseudomonas syringae* as a second pathosystem. Our results indicate an interaction between transcriptomic responses to N and biotic stress. A small fraction of transcripts was prioritized between antagonistic responses, reflecting a preservation of the plant defense program under N limitation. Furthermore, this interaction also led to a complex and specific response in terms of metabolism and cellular homeostasis-associated genes.

## 1. Introduction

Sustainable protection of crops is a worldwide necessity. Indeed, plants are often continuously exposed to a broad range of biotic and abiotic stresses in their natural habitats [1]. Because biotic and abiotic stresses significantly reduce plant growth and productivity, considerable research has aimed to determine the responses of plants to single stresses [2]. However, the response of plants to a combination of stresses is not the simple addition of the response to each single stress [3]. In addition, it has also been reported that plant responses to different stresses are coordinated by complex and interconnected signaling pathways modulating numerous metabolic networks [4]. Apart from some recent reports, the effects of combined biotic and abiotic stress have been little studied. Indeed, various abiotic stresses can negatively or positively affect plant–pathogen interactions. For example, the exposure of Arabidopsis plants to drought enhances plant susceptibility to an avirulent isolate of *Pseudomonas syringae*, while it decreases the susceptibility of tomato to the fungus *Botrytis cinerea* [5]. One of the most important abiotic factors is the availability of nutrients that seriously affects plant disease severity [6]. Contradictory reports about the impact of nutrients on plant susceptibility to pathogens indicate that many factors affecting this process are not well understood. 

Nitrogen (N) is an essential macronutrient and a major limiting factor of plant growth and development [7]. In addition to growth and developmental effects, many agronomical reports highlight the fact that N fertilizers can impact the plant’s ability to cope with biotic stress [8]. However, contradictory data have been reported about the effect of N availability on disease development. On the one hand, the effect of N availability on this process seems partly dependent to the lifestyle of the pathogen. Generally, high N availability increases the susceptibility of plants to biotrophs, while it generally decreases the susceptibility of plants to necrotrophs, although some exceptions have been reported [9]. However, this process seems to be more complex and the impact of N availability can be dependent on the host plant for a given pathogen. For example, in the case of *B. cinerea*, one of most important fungal plant pathogens, high N fertilization enhances infection in strawberry [10], while it reduces susceptibility to this fungus in tomato [11]. The form of N available can also determine the effect of N supply on disease severity. For instance, NH_4_^+^ fertilization enhances the level of symptoms caused by *P. syringae*, while, conversely, NO_3_^−^ reduces plant susceptibility to *P. syringae* [12]. These studies indicate the complexity of the relationship between N metabolism and plant resistance to pathogens.

Although the mechanisms are not well known, it is generally thought that N supply can affect plant–pathogen interactions through its impact on plant defense, pathogen virulence, and the availability of nutrients for the pathogen [9]. However, the effect of N limitation on the expression of defense remains unclear, as the literature harbors contradictory reports concerning the effect of N limitation on the expression of defense. Indeed, some reports highlight that N limitation can influence constitutive or induced defense [13]. Many reports show that limiting N supply drives a decrease in defense [14]. Indeed, Arabidopsis plants grown in low N supply show a reduced basal activity of three defense-associated enzymes (chitinase, chitosanase, and peroxidase). Furthermore, in plants grown in low N and treated with BION^®^, a chemical elicitor of plant defense, the level of these enzymes is also reduced, compared to plants grown in high N [15]. Recently, it was demonstrated that under low N, there is a decrease in the levels of polyamines, compounds known to increase plant resistance via the triggering of programmed cell death [16,17]. However, other reports suggest that there could be a trade-off between plant growth and defense [18]. Thus, although it is obvious that N availability has an impact on plant defense, it is currently difficult to get a clear-cut idea of the effect of N availability on plant defense.

*Erwinia amylovora* (*Ea*) is the bacterial phytopathogen responsible for fire blight disease. *Ea* is a major concern for apple and pear orchards worldwide, as there is no genetic source of complete resistance. We showed previously that *Ea* can multiply in Arabidopsis and that N supply affects the susceptibility of Arabidopsis to *Ea* [9]. In this paper, we studied the impact of N limitation on the response of Arabidopsis to *Ea*. We analyzed the transcriptome of plants grown in low and full N and subjected to biotic stress. Altogether, our data suggest a preponderance of the plant’s response to biotic stress in terms of genes modulation over the response to N limitation. In order to determine the genericity of the effect of N supply on biotic stress, we analyzed the impact of N supply on the response of selected defense marker genes to a virulent and an avirulent strain of the bacterial phytopathogen *P. syringae*.

## 2. Results

### 2.1. Effect of N Limitation on Gene Expression in Response to Erwinia amylovora (Ea)

**Figure 1:**
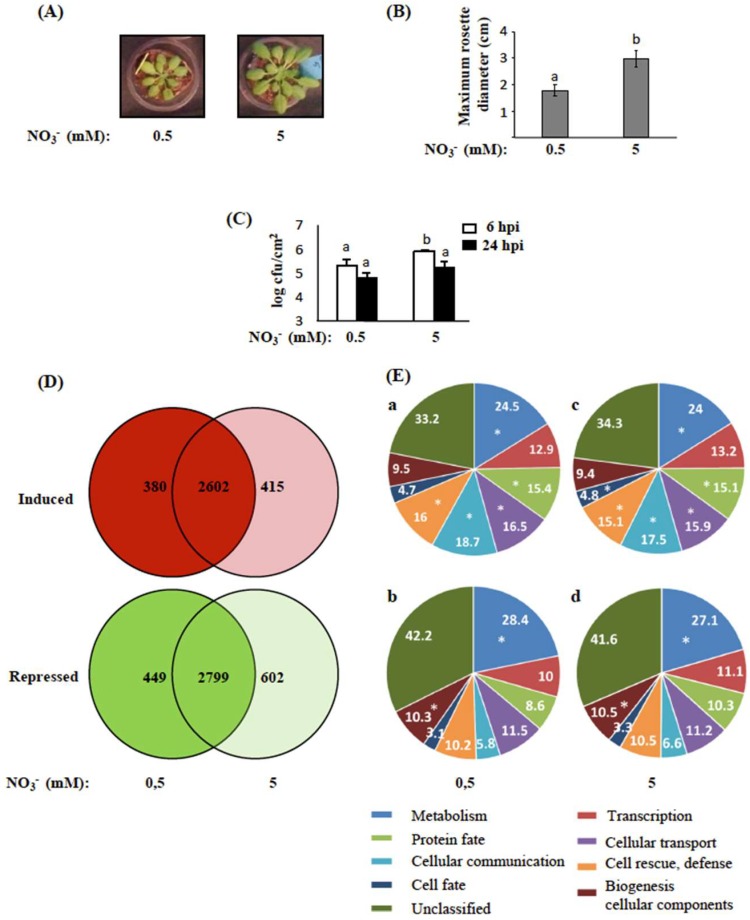
Impact of nitrogen (N) on physiological and transcriptional characteristics of Arabidopsis plants. (**A**) Five-week-old Arabidopsis rosettes grown under low and full N. (**B**) Maximum rosette diameter quantified by ImageJ. (**C**) Bacterial cell numbers of *Erwinia amylovora* (*Ea*) CFBP1430 in Arabidopsis rosette leaves at 6 and 24 h post inoculation (hpi). (**B**,**C**) Different letters indicate significant differences according to the Mann–Whitney test (*p*-value < 0.05). All experiments were repeated twice or more with similar results: For (**B**), the results of two independent experiments were pooled (*n* = 20); for (**C**), a representative experiment is shown (*n* = 3). (**D**,**E**) Transcriptomic analysis of Arabidopsis plants grown in contrasted N regimes and inoculated with *Ea*. Plants were sampled 6 hpi; two biological replicates were performed for each condition. (**D**) Venn diagrams illustrating the overlap of upregulated (**red**) and downregulated (**green**) Arabidopsis genes in response to *Ea* between low and full N. (**E**) Distribution of functional categories according to the FunCatDB. The pie charts represent: (**a**) 2982 genes induced and (**b**) 3248 genes repressed by *Ea* in plants grown in low N; (**c**) 3017 genes induced and (**d**) 3401 genes repressed by *Ea* in plants grown under full N. Asterisks (*) indicate significant differences according to hypergeometric distribution (*p*-value < 0.05).

To determine to what extent the nutritional status of a plant affects its response to a bacterial pathogen, we grew Arabidopsis plants in low (0.5 mM) or full (5 mM) NO_3_^−^ in plugs of peat moss substrate for 5 weeks (Figure 1A), and infected them with the bacterial phytopathogen *Ea*. In these conditions, the rosette diameter was reduced by 38% (Figure 1B) and nitrate content was reduced by 16% (Figure S1), indicating that NO_3_^−^ was limiting for plant growth in the 0.5 mM NO_3_^−^ condition compared to 5 mM NO_3_^−^ (full N). In order to determine if plant susceptibility to bacteria was affected by these growth conditions, plants were inoculated with *Ea* wild-type strain and in planta bacterial cell numbers was analyzed 6 and 24 h post inoculation (hpi). Bacterial cell numbers were lower in leaves of Arabidopsis plants grown in low N compared to those grown in full N (Figure 1C).

In order to test the hypothesis that lower in planta bacterial cell numbers are due to a difference in the expression of defense in plants grown in low N, we analyzed the transcriptome of plants grown in low and full N and infected or not with *Ea*. Leaves of five-week-old plants were *Ea-* or mock-inoculated and sampled 6 hpi. Gene expression was analyzed using the CATMA microarray. We first compared gene expression in noninfected plants to a previous microarray dataset from Arabidopsis plants grown in low nitrate [19]. As expected, several genes previously described as repressed in low nitrate conditions, such as *ASN2* [20], were less expressed in plants grown in low N than in plants grown in full N. Then, log2 of the ratio between expression in infected and mock plants was calculated. In order to validate our dataset, we compared our data to previous transcriptomic analysis performed in response to *Ea* [21] and found that previously identified *Ea*-responsive genes showed, for the most part, a similar profile in the present dataset (Figure S2). The Pearson correlation coefficient between the two sets of data was high (*R*^2^ = 0.95; Figure S3), indicating that response to infection of plants grown in contrasted N supply conditions was very close. Statistical analysis of the data indicated that the expression of 2982 and 3017 genes was significantly induced (Bonferroni, *p*-value < 0.05) following infection with bacteria, in plants grown in low and full N, respectively. Altogether, 2602 genes showed an increase in expression following infection in both N regimes (Figure 1D). We also found that 3248 and 3401 genes were significantly repressed (Bonferroni, *p*-value < 0.05) following *Ea* infection in plants grown in low and full N, respectively. 2799 genes showed a decrease in expression following infection in both N conditions (Figure 1D). Several hundreds of genes were modulated in response to *Ea* only in one N condition (Figure 1D). However, only a small number of these genes, described later, showed a strong difference in expression profile between the two N regimes (Table S1 and Figure S3).

We then analyzed the functional categories of the infection-modulated genes using the Munich Information Center for Protein Sequences database [22]. For *Ea*-induced and *Ea*-repressed genes, the main functional categories represented were found both for plants grown in low and full N (Figure 1E), which is not surprising, given the similarity of the datasets obtained for plants grown at in low and full N (Figure S3). The largest categories represented unclassified and metabolism-related genes (Figure 1E). Several functional categories were significantly overrepresented in our datasets compared to their representation in the whole genome (categories with an asterisk in Figure 1E). In most cases, their overrepresentation was different between *Ea*-induced and *Ea*-repressed genes, but was not affected by the N regime of the plants. Only the “cell fate” category, which mainly contains genes associated with cell growth, showed a difference according to the NO_3_^−^ level, as it was significantly overrepresented only in plants grown in full N for bacteria-induced genes. Among bacteria-induced genes, the “metabolism”, “protein fate”, cellular transport”, “cellular communication”, and “cell rescue and defense” functional categories were overrepresented. Among bacteria-repressed genes, the “metabolism” and “biogenesis of cellular components” categories were overrepresented. These categories are consistent with the response of the plant to biotic stress, which is known to lead to strong metabolic readjustment, defense setup and a shutting down of the photosynthesis apparatus.

Our data indicate that the very large transcriptional reprogramming previously observed in Arabidopsis leaves infected with *Ea* [21] occurs whether plants are grown in low or full N. Despite the strong impact of the N limitation on the growth of the plants, these plants showed a response to biotic stress that was mostly similar to plants grown in full N, with only strong differences in expression for a small subset of genes.

### 2.2. N Availability Modulates Specific Defense-Related Genes

Little is known on the impact of plant nutrition on the expression of genes following biotic stress. To analyze the impact of N supply on known defense-associated gene expression, we compared the gene expression ratio (log2) between infected and mock plants grown in low or full N (Table 1). Most Ethylene- (ET) and Salicylic acid (SA)-related genes analyzed were highly induced by *Ea* infection in plants grown in low and full N. In general, the level of modulation by infection of the SA- and ET-associated genes analyzed here was not strongly affected by N availability. However, two SA-responsive genes, *PR2* and *PR5*, were induced only in plants grown upon N limitation (Table 1). Conversely, most Jasmonic acid (JA)-associated genes were either not modulated following bacterial infection or were repressed by bacteria. However, we found that two genes involved in JA biosynthesis, *AOS* (allene oxide synthase) and *AOC2* (allene oxide cyclase), and the JA-responsive gene *JR1* (Jacalin lectin family protein) were repressed only in plants grown in full N. Only the JA-responsive *PR4* gene was induced by *Ea*, and this was restricted to plants grown under low N conditions. Altogether, these data indicate that N availability modulates, at least in part, the known plant defense response to pathogens, with a strong impact on the JA-signaling pathway. 

We also analyzed the impact of N supply on the response to bacterial infection of known defense-related regulator genes. For example, WRKY transcription factors (TF) are well known regulators of the biotic stress response in plants. The majority of WRKY TFs were indeed modulated by *Ea* infection, but in most cases, there was no impact of N supply on this modulation (Table S2). Only five WRKY TFs showed a differential modulation in response to bacteria under different N regimes (Table S2): Four were induced only in full N (*WRKY42, WRKY47, WRKY64, WRKY67*) and two were repressed only in full N (*WRKY3* and *WRKY69*). Concerning MYB TFs, which have been in some cases associated with the defense response [23], most genes were not modulated by *Ea* or repressed independent of the N regime. Only three genes showed an N-regime specific profile (AT1G25550, AT5G17300 and AT1G74840). 

We hypothesized that the genes showing the strongest difference in the amplitude of the modulation by bacterial infection could be good candidates to explain the difference in susceptibility to *Ea* of Arabidopsis plants grown under different N regimes. We thus looked for genes with the highest differential in log2 ratio in response to infection between plants grown in low and full N. The thirty genes with the highest differential in log2 ratio showed mostly two types of profiles (Table S1): Genes repressed by bacterial infection in low N but not in full N, and genes induced by bacterial infection specifically or more highly in low N. Interestingly, genes which were repressed by bacteria only in low N were mainly associated to metabolism (Table S1), while genes which were more or only induced in low N were mainly linked to plant defense responses. For instance, two kelch repeat-containing F-box family proteins (AT1G80440, AT2G44130) were involved in the regulation of the phenylpropanoid biosynthesis pathway [24], an ankyrin repeat protein (AT5G54610) was involved in regulation of immunity [25], and a leucine-rich repeat family protein (AT3G11010) was involved in defense signaling [26].

### 2.3. N Limitation and Biotic Stresses Interact

**Figure 2:**
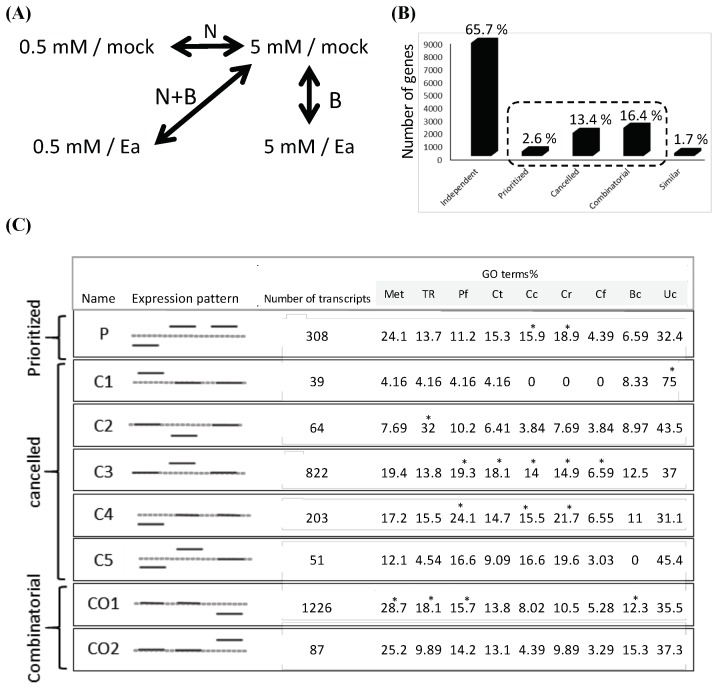
Gene expression patterns in response to single and combined stresses. (**A**) Schematic representation of single stresses (N:N limitation; B: *Ea* infection) and combined stresses (N + B:N limitation and *Ea* infection); arrows represent the dataset comparisons performed that are represented as “expression pattern” in (C). (**B**) Number of genes in the different categories of responses to stress combination; for each category, the percentage among the total modulated genes is indicated. The dotted line indicates the three nonpredictable categories, which represent one third of *Ea*-responsive transcripts. (**C**) Detail of the expression patterns among the different nonpredictable categories. Expression patterns: The dotted line represents transcript level in control plants (mock/5 mM NO_3_^−^); the full lines correspond to N, B, and N + B (from left to right). For each subcategory, only the type of response is indicated with a full line (induced, repressed, no response). Enrichment in Gene ontology (GO) terms in each subcategory was determined according to the FunCatDB. Asterisks (*) indicate a significant difference relative to the Arabidopsis genome (*p*-value < 0.05)*.* P: Prioritized, C: Cancelled, and CO: Combinatorial. Met: Metabolism, Tr: Transcription, Pf: Protein fate, Ct: Cellular transport, Cc: Cellular communication, Cr: Cell rescue, defense, Cf: Cell fate, Bc: Biogenesis of cellular components, Uc: Unclassified.

In order to test the hypothesis of an interaction between the response of plants to N limitation and the response to biotic stress, we analyzed the response of plants to the combination of stresses in more detail. For this, we compared each single stress (N limitation or bacteria) to the combination of the two stresses (Figure 2A). We used the categories defined by the authors of [1] to classify the genes according to their pattern of expression, using a log2 (ratio) of 1 as a cutoff. Furthermore, the responses were considered in a simplified fashion as “induced/not modulated/repressed”, without considering the level of expression. Comparison of the responses of single versus combined stresses showed that around two thirds of the genes modulated in our experiments show an independent response (Figure 2B). Indeed, these genes show a response to the stress combination (N + B) that corresponds to the response of the gene to one of the single stresses. This was expected, since the overlap between infection-modulated genes between plants grown in low and full N is very high (Figure 1D). However, one third of the genes showed a response to the combination of the stresses that could not be deduced from their response to each individual stress, suggesting an interaction between the two single stress responses. These nondeducible gene patterns fall into three categories, defined previously [1], which are combinatorial, cancelled, and prioritized (Figure 2C). Only a few transcripts responded in the prioritized manner (Figure 2C). The prioritized category corresponds exclusively to genes that are repressed by N limitation, induced by biotic stress in full N conditions and that remain induced by biotic stress in low N conditions. The majority of the genes showing a nondeducible pattern showed either a combinatorial pattern (16.4% of total modulated genes) or a cancelled pattern (13.4%, of total modulated genes). Furthermore, the expression profiles corresponded to a small number of specific expression patterns (Figure 2C). In the cancelled category, we found five subcategories (C1–C5; Figure 2C) corresponding to genes induced or repressed by one of the single stresses and not modulated in response to the combination. Among these five categories, the most abundant correspond to genes induced by biotic stress and not modulated in response to the combination (C3) and genes repressed by N limitation and not modulated in response to the combination (C4). The most surprising category corresponds to the combinatorial pattern (Co1–Co2; Figure 2C). These genes correspond to genes that are not modulated in response to single stresses, but that show repression (the most abundant subcategory; Co1) or induction (Co2) in response to the combination.

We then determined whether transcripts of each specific response mode could be linked to particular biological functions (Figure 2C). The prioritized mode was primarily associated to “cell rescue, defense” and “cellular communication”. In the canceled and combinatorial categories, the largest subcategories (C3 and Co1) corresponded to a more diverse set of functional categories. In the Co1 pattern, the “metabolism” was very important, while in the C3 category, the “protein fate and cellular transport” functional categories were very important (Figure 2C). This may reflect a complex and specific adaptation of the plant to the combination of stresses.

### 2.4. Defense-Associated Genes Are Prioritized in Response to the Combination of N Limitation with Different Pathogens

Since our analysis of the transcriptome data indicated that defense-associated genes were overrepresented in the prioritized mode, we analyzed the expression profile of specific defense-associated genes known to be expressed in response to biotic stress. We found a large number with a prioritized pattern. This is particularly true for the WRKY family of transcription factors [27] that have largely been described as being involved in the response of plants to stress (Table 2). Another typical defense gene, *PR1*, was also found to be regulated in the prioritized mode. We compared these results to N metabolism-associated genes (Table 2). Most N-related genes showed profiles that were independent in the stress combinations (Table S3), including three members of the NRT2 family of putative nitrate transporters, *NRT2.1* and *NRT.6*, known to be involved in the response to biotic stress [28,29]. Only five genes related to N metabolism showed a specific profile in response to the combination of the two stresses (Table 2). Three of these genes showed a prioritized pattern, while two showed a cancelled pattern. Interestingly, the three genes showing a prioritized pattern have been linked to defense responses. Indeed, *Lysine Histidine Transporter 1* (*LHT1*) and *Ammonium Transporter 1* (*AMT1*) have been shown to be involved in defense against pathogens [30,31] while *Wound-responsive gene 3* (*WR3/NAR1*), encoding a component of the high-affinity nitrate transporter system, is involved in JA-independent wound signal transduction [32]. Thus, our data show that genes known to play a role in Arabidopsis defense against pathogens were regulated in a prioritized manner, which is consistent with the fact that N limitation affected the response of the plant to biotic stress only for a limited subset of genes (Figure 2).

**Figure 3:**
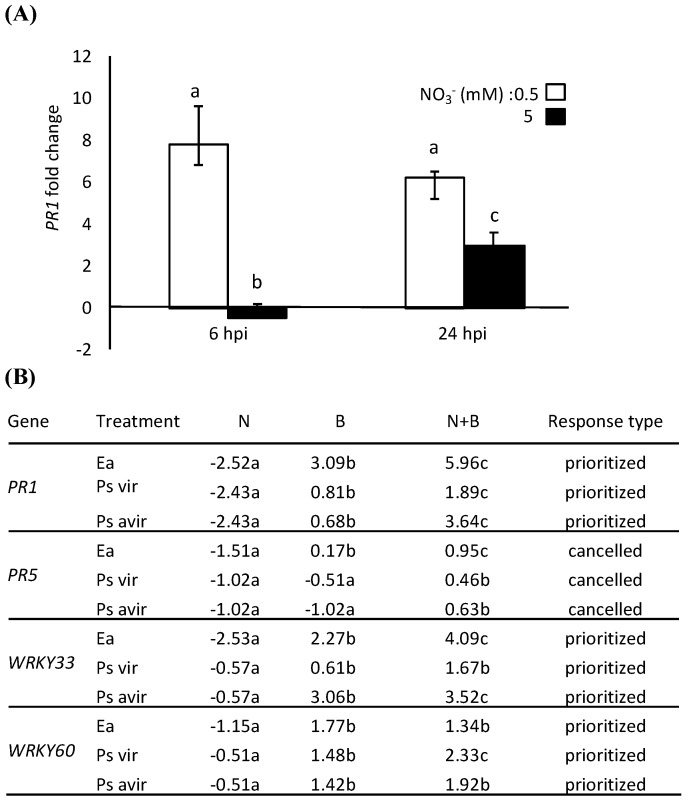
Impact of N supply on defense-related genes expression in response to different bacterial pathogens. (**A**) Time-course of *PR1* gene expression at 6 and 24 hpi following mock or *Ea* treatment. (**B**) Response of selected defense-related genes in response to N, B, and N + B at 6 hpi. (**A**,**B**) Col-0 plants were grown in full (5 mM) or low (0.5 mM) NO_3_^−^. and infiltrated with bacteria (*Ea* wild type, virulent and a virulent strain of *P. syringae*) or mock-inoculated. Expression is normalized to the *PP2a3* (At1g69960) constitutive gene. Values are log2 signal ratios between infected and mock plants. Similar results were obtained in a minimum of three independent experiments, including three biological replicates each; a representative experiment is shown. The bars represent standard deviation. (**A**,**B**): Different letters correspond to values that are significantly different according to the Mann–Whitney statistical test (*p*-value < 0.05).

In order to determine how generic the interaction between stresses is, we analyzed the effect of N limitation on the response of Arabidopsis to another bacterial phytopathogen, *P. syringae* pv. *tomato.* We used the virulent strain DC3000 and the avirulent strain DC3000 *avrrpm1*. Arabidopsis plants were grown for five weeks in peat moss in low N or full N, as described above. After 5 weeks, rosette leaves were inoculated with the virulent or the avirulent strain of *P. syringae*. As a control, we also inoculated plants with *Ea*. In order to study the kinetic of gene expression, plants were harvested at 6 and 24 hpi and gene expression was analyzed by qRT-PCR as above. We selected three defense-related genes, *PR1, WRKY33* and *WRKY60*, which exhibited a prioritized response and one, *PR5*, which exhibited a cancelled response. Interestingly, we not only found that *PR1* was more highly expressed in response to *Ea* in plants grown under low N, but also that *PR1* was induced by *Ea* as early as 6 hpi only in plants grown in full N (Figure 3A). Furthermore, qRT-PCR analysis of these genes confirmed the prioritized response of these genes in combination of N limitation and *P. syringae*, both for the virulent and the avirulent strain tested (Figure 3B).

Our data show that defense-related genes respond in the same manner when N limitation is combined to different bacterial pathogens.

## 3. Discussion

Several studies have demonstrated that the availability of nutrients, in particular of nitrogen (N), influences the outcome of plant–pathogen interactions. Nevertheless, the mechanisms underlying this connection are poorly understood, in part because the effect of N availability on this biotic stress is dependent on the plant–pathogen interaction considered [6,9,14,33]. Therefore, it is currently difficult to define general rules for the impact of N availability on the response of plants to biotic stress. Previous reports have suggested that defense activation in plants grown under N limitation is reduced [15]. However, these data concerned a limited number of defenses and this did not allow to determine whether N limitation affected the response of a plant to biotic stress on a large scale. More recently, a transcriptomic analysis showed that the response of tomato to the fungus *B. cinerea* is affected by N supply [11]. However, data concerning combinations of biotic and abiotic stresses at the transcriptomic level remain scarce [27] and no study has analyzed the impact of N supply on the response to biotic stress in Arabidopsis.

Bacterial infection leads to large modifications in the transcriptomic profile of Arabidopsis plants at early time-points post inoculation [30,31]. This was shown for virulent and avirulent *P. syringae* strains, the response to which mainly differs in timing and intensity [32], as well as for necrotrophic bacterial pathogens, such as *Ea* [6,9,14,33]. To test the hypothesis that the response of plants to *Ea* infection is affected by N limitation, we analyzed the transcriptome of rosette leaves of plants grown in low or full N and exposed to the phytopathogenic bacterium *Ea*. Our data indicated, as expected, a large transcriptomic reprogramming following plant infection by *Ea*, with an important induction of defense-associated genes in both low and full N growth conditions. Close examination of the transcriptomic data indicated a large overlap in the plants’ response to bacteria in plants grown in low or full N. Indeed, in our dataset, there was an 86% overlap between genes modulated in low and full N, suggesting that, at least at the qualitative level, the response of plants to biotic stress was close in low and full N. This was rather surprising given the important impact of N limitation on plant size in our experiments. Indeed, the commonly held idea of a tradeoff between plant growth and plant defense has led to the widespread idea that plants have a lower capacity to defend themselves when growth is optimal, thus when nitrogen is abundant [34].

We further analyzed our transcriptome data in response to single (N limitation or bacteria) and combined (N limitation and bacteria) stresses, according to a previous analysis of transcriptomic data performed on several combinations of stresses [15]. As in this previous study, we found that three categories (independent, combinatorial, and cancelled), among the five types of expression profiles identified, represent the most abundant transcript response modes (>95% of the total transcripts). Furthermore, as in this previous study, the deducible profiles (independent and similar) were much more frequent than the nondeducible profiles (combinatorial, canceled, and prioritized), which constituted a third of the total transcripts. In addition, we found that the response of plants to the combination of stresses was closer to its response to bacterial treatment than to N limitation alone. This reflects a dominance of the response to biotic stress over the response to the abiotic stress in our experiments. On the other hand, a recent transcriptomic study on Arabidopsis response to sequential double stresses indicated that plants first subjected to drought or herbivory stress and then infected by *B. cinerea* responded similarly to *B. cinerea* treatment alone. The authors proposed that when two stresses are applied in sequence, plants display a transcriptome profile, which is very similar to the second stress, regardless of the nature of the first stress [11]. Since our experimental setup involves applying N limitation before bacterial infection, one could imagine that the dominance of biotic stress is, at least partially, a result of the experimental setup.

The prioritized category corresponded exclusively to genes repressed by N limitation and induced by bacteria and combined stress. The “cell rescue, defense” was overrepresented among these genes. This indicates that signaling pathways regulating defense against bacteria are negatively regulated by abiotic stress. Previous studies showed that defense genes activated by *B. cinerea* were repressed under drought stress [35], suggesting that repression of defense expression occurs in response to different abiotic stresses and prioritized in response to different pathogens. We confirmed the prioritized mode for some selected defense genes in response to N limitation and another bacterial phytopathogen, *P. syringae*. The expression of N-related genes has been described to be affected by bacterial infection and recent reports show that some of these genes are indeed involved in plant defense responses to pathogens [1,8,11,36]. Our transcriptomic data indicated that several genes related to N metabolism, such as *LHT1* and *NRT2.6,* were strongly induced by bacteria. Interestingly, we found that these genes showed a prioritized profile, like many defense genes, and not an independent mode, like most N-associated genes. This suggests that although these genes are involved in N metabolism and/or transport, they are regulated by the plant as defense genes independently of N supply. On the other hand, *NRT2.1* was upregulated by bacteria only in plants grown in full N. Interestingly, a recent transcriptomic study on tomato indicated that *NRT2.1* is also upregulated by *B. cinerea* only in plants grown in full N [11]. This suggests that induction of *NRT2.1* is N-dependent in response to different pathogens, suggesting a conserved mechanism. *NRT2.1* has been shown to be a down-regulator of salicylic acid-dependent defenses in response to *P. syringae*. These results suggest that *NRT2.1* could play a role in negatively controlling defense activation in response to pathogens in full N in different pathosystems.

Infection with *Ea* activates defense-related hormonal signaling pathways. The SA-signaling pathway is indeed strongly induced following *Ea* infection in a T3SS-dependent manner, both in host and nonhost plants [37,38]. Regulation of the JA-signaling pathway in response to *Ea* infection seems more complex. Indeed, several genes involved in JA biosynthesis are repressed following *Ea* infection in Arabidopsis, but other JA-dependent genes are induced following infection by *Ea* ([21] and this study). In parallel, it was shown that T3SS-dependent downregulation of the JA pathway is a critical element in the infection process of Malus spp. by *Ea*, since the addition of methyl-jasmonate to susceptible plants increases their resistance to *Ea*. In contrast, the SA pathway was similarly induced in both resistant and susceptible Malus spp by *Ea* [39]. Study of hormonal pathway related genes in our transcriptomic data indicated that SA- and ET-associated genes were highly induced by *Ea*, while most of the JA-related genes were repressed by *Ea*. Generally, in response to *Ea*, the ET and SA pathways were not significantly affected by N supply; however, some genes involved in JA biosynthesis and JA-responsive genes were repressed only in plants grown in full N, suggesting that N supply modulates plant–pathogen interaction through the JA pathway signaling. Thus, the higher bacterial cell numbers in plants grown in full N could be linked to higher repression of JA-associated defense in these plants. Interestingly, these results are reminiscent of those observed with the *B. cinerea*–tomato interaction, for which expression of the JA pathway was clearly identified as being associated with the lower symptoms observed in plants grown in high N [40].

## 4. Materials and Methods

### 4.1. Growth Conditions of Arabidopsis Plants

Seeds of *Arabidopsis thaliana* Col-0 were obtained from the INRA Versailles collection. Plants were grown for 5 weeks in 4 cm plugs of peat moss substrate (70% blond peat, 20% perlite, and 10% vermiculite) wrapped in a nonwoven film [41] and were subjected to an 8 h-light (150 μmol·m^−2^·s^−1^ irradiation) and 16 h-dark cycle at 21 °C (day)/18 °C (night), with 65% relative humidity. Nitrogen limitation was performed as described in Reference [9]. Briefly: Plants were grown for five weeks in soil and watered to a final humidity of 60%, with a nutrient solution containing full (5 mM NO_3_^−^) or low nitrogen (0.5 mM NO_3_^−^). In all cases, 5-week-old stressed or control plants were mock or pathogen-inoculated as described in the text.

### 4.2. Pathogen Infections

Rosette leaves of 5-week-old plants were infiltrated with *Ea* CFBP1430 using a needleless syringe. Bacterial suspensions were prepared in sterile water (10^7^ CFU·mL^−1^). Six and twenty-four hours after infection (hpi), we performed bacterial counting by grinding infected leaves using glass beads in a TissueLyser (Qiagen/Retsch, Hilden, Germany). The bacterial suspensions were used to prepare serial dilutions, which were plated on an LB medium, and after 1 or 2 days the colonies formed were counted to evaluate the initial number of bacteria.

### 4.3. RNA Isolation and qRT-PCR Analysis

For RNA extraction, twelve leaves of three plants (pathogen- or mock-treated) were collected at the indicated time-point after treatment, pooled, and immediately frozen in liquid nitrogen. The experiment was repeated twice independently, thus *n* = 6. Total RNA was extracted from 100 mg of frozen ground leaves using Trizol^®^ reagent (Invitrogen Life Technologies, Saint-Aubin, France). RNA quality was evaluated by electrophoretic run on 1% agarose gel. For the qRT-PCR analysis, first-strand cDNA was synthesized using Superscript reverse transcriptase SSII (Invitrogen, Saint-Aubin, France) from 1 μg of DNase-treated (Invitrogen) total RNA in a 20 μL reaction volume. qPCR reactions were performed using SYBR^®^ Selected MasterMix 2x (Applied Biosystem, Villebon Sur Yvette, France), following the manufacturer’s protocol. The cycling conditions consisted of an initial 5 min at 95 °C, followed by 40 three-step cycles at 94 °C for 15 s, 60 °C for 30 s, and 72 °C for 30 s. Melting curve analysis was performed after cycle completion to validate amplicon identity. Relative expression levels were calculated following the standard curve based method [42]. Expression of the *Protein Phosphatase 2a Subunit A3* (*PP2A3*) reference gene [43] was used for normalization of every target gene studied. For each treatment, three biological replicates, corresponding to a pool of 4 leaves from a single plant, were analyzed and each qRT-PCR reaction was carried out in duplicate; the complete experiment was conducted twice independently and one representative experiment is presented in Figure 3. The gene-specific primers used in this analysis are indicated in Table S4.

### 4.4. Transcriptome Studies

Microarray analysis was carried out at the Institute of Plant Sciences Paris-Saclay (IPS2, Evry, France), using the CATMAv7 array [34] based on AGILENT technology. The CATMAv7 design of *Arabidopsis thaliana* genome was made with gene annotations included in FLAGdb++ (http://tools.ips2.u-psud.fr/FLAGdb), an integrative database around plant genome [1]. The single high density CATMAv7 microarray slide contains four chambers, each containing 149916 primers. Each 60 bp primer is triplicate in each chamber for robust analysis and in both strands. As part of all probes, 35,754 in triplicate correspond to gene TAIRv8 (among which, 476 probes correspond to mitochondrial and chloroplast genes), together with 1289 probes corresponding to EUGENE software predictions and 658 probes for miRNA/MIR, and finally 240 controls. Two independent biological replicates were produced. For each biological replicate, RNA samples were obtained by pooling RNAs from more than three leaves. Leaves were collected on plants at 3.90 developmental growth stages [35] cultivated in short day conditions. Total RNA was extracted using Trizol^®^, followed by a purification step on RNeasy column (Qiagen, Courtaboeuf, France) according to the supplier’s instructions. For each comparison, one technical replicate with fluorochrome reversal was performed for each biological replicate (i.e., four hybridizations per comparison). The labeling of cRNAs with Cy3-dUTP or Cy5-dUTP was performed as described in Two-Color Microarray-Based Gene Expression Analysis Low Input Quick Amp Labeling manual (©Agilent Technologies, Inc., Les Ulis, France). The hybridization and washing were performed according to Agilent Microarray Hybridization Chamber User Guide instructions (©Agilent Technologies, Inc.). Two-micron scanning was performed with InnoScan900 scanner (InnopsysR, Carbonne, France) and raw data were extracted using MapixR software (version 7.1.0, InnopsysR, Carbonne, France).

Microarray data from this article were deposited in the international repository GEO, Gene Expression Omnibus (Edgar R. 2002, http://www.ncbi.nlm.nih.gov/geo/), accession No. GSE97582) and all steps of the experiment, from growth conditions to bioinformatic and statistical analyses, were detailed in CATdb [28,29] (http://tools.ips2.u-psud.fr/CATdb/; Project: RA14-05_Multipass) according to the “Minimum Information About a Microarray Experiment” standards.

### 4.5. Statistical Analysis of Microarray Data

Experiments were designed with the Genomic networks team of IPS2. For each array, the raw data comprised the logarithm of median feature pixel intensity at wavelengths 635 nm (red) and 532 nm (green). For each array, a global intensity-dependent normalization using the loess procedure [37,38] was performed to correct the dye bias. The differential analysis is based on the log-ratios averaging over the duplicate probes and over the technical replicates. Hence, the number of available data for each gene equals the number of biological replicates, and these were used to calculate the moderated *t*-test [39].

Under the null hypothesis, no evidence that the specific variances vary between probes was highlighted by Limma and, consequently, the moderated *t*-statistic was assumed to follow a standard normal distribution. To control the false discovery rate, adjusted *p*-values found using the optimized FDR approach [44] were calculated. We considered as being differentially expressed the probes with an adjusted *p*-value ≤ 0.05. Analysis was done with the R software (R Development Core Team, 2005, https://cran.r-project.org). The function SqueezeVar of the Limma library was used to smooth the specific variances by computing empirical Bayes posterior means. The library kerfdr was used to calculate the adjusted *p*-values.

### 4.6. Transcriptional Response Modes

Transcript sets were created by grouping genes exhibiting similar expression patterns under single (N limitation or bacteria) and combined stress (N limitation and bacteria) treatments. The responses were considered in a simplified fashion as “induced/not modulated/repressed”, without taking into account the level of expression: Log-fold changes between −1 and 1 were considered as nonregulated genes, and log-fold changes higher than 1 and lower than −1 were considered as induced and repressed genes, respectively (Table 2). Plant response to N limitation corresponds to ratios (log2) between control plants grown in low (0.5 mM) and full (5 mM) NO_3_^−^; plant response to bacteria corresponds to ratios (log2) between infected and control plants grown in full (5 mM) NO_3_^−^; plant response to combined stresses corresponds to ratios (log2) between infected plant grown in low (0.5 mM) NO_3_^−^ and control plants grown in full (5 mM) NO_3_^−^.

Genes were grouped into 20 subcategories, each representing a specific expression pattern. The 20 subcategories were assembled into five larger categories (cancelled, combinatorial, prioritized, independent, and similar) according to [1]. GO terms associated with each specific transcriptional response profile using the FunCatDB [22].

### 4.7. Data Availability

The datasets generated and analyzed during the current study are available in the CATdb repository: http://tools.ips2.u-psud.fr/cgi-bin/projects/CATdb/consult_project.pl?project_id=402.

## 5. Conclusions

Our work shows that while a large proportion of the Arabidopsis genes showed the same modulation profile (induced or repressed) in response to bacterial infection in two contrasted N regimes, a small number of transcripts showed specific responses to the combination of stresses, including known defense-related genes, thus maybe reflecting a preservation of the plant defense program under N limitation treatment. We found that N limitation had a similar effect on defense gene expression in response to two different bacterial pathogens, indicating at least partial conservation of the interaction between the response to biotic stress and to N limitation. Finally, our work suggests a main role for the JA-signaling pathway in the impact of N supply on the response of plants to biotic stress, as suggested by a previous report [11]. It is probably of great interest to further study this link in future work.

## Figures and Tables

**Table 1:** Effect of N limitation on defense-related genes. Values are log2 signal ratios between infected and water-treated control plants. BS: Genes involved in hormone biosynthesis, R: Hormone-responsive genes, S: Genes involved in hormone signaling. Two levels of significance threshold were considered according to the Bonferroni statistical test (a: *p*-value < 0.05; b: *p*-value < 10^−8^).

Gene Number	Name	Function	Low N		Full N	
			*Ea* vs. Mock		*Ea* vs. Mock	
SA biosynthesis and responsive genes						
*AT1G74710*	*ICS1*	BS	6.14	b	5.73	b
*AT3G52430*	*PAD4*	BS	5.15	b	4.51	b
*AT3G48090*	*EDS1*	S	3.85	b	3.82	b
*AT4G39030*	*EDS5*	S	6.33	b	5.67	b
*AT1G64280*	*NPR1*	S	2.14	a	1.66	a
*AT2G14610*	*PR1*	R	5.10	b	3.23	b
*AT3G57260*	*PR2*	R	1.50	a	0.03	
*AT1G75040*	*PR5*	R	2.00	b	0.88	
ET biosynthesis and responsive genes						
*AT1G05010*	*ACO*	BS	2.64	b	1.62	a
*AT3G04580*	*EIN4*	R	2.76	b	2.66	b
*AT4G17500*	*ERF*-*1*	R	1.93	a	1.92	a
*AT2G40940*	*ERS1*	R	1.42	a	1.05	a
*AT1G66340*	*ETR1*	R	1.19		1.14	
JA biosynthesis and responsive genes						
*AT3G25770*	*AOC2*	BS	−1.17		−2.12	b
*AT5G42650*	*AOS*	BS	−1.13		−2.13	b
*AT2G46370*	*JAR1*	BS	−0.23		−0.51	
*AT3G45140*	*LOX2*	BS	−0.35		−0.97	
*AT2G06050*	*OPR3*	BS	0.16		0.26	
*AT1G19640*	*JMT*	R	−0.60		−0.23	
*AT3G16470*	*JR1*	R	0.08		−2.01	a
*AT2G26020*	*PDF1.2b*	R	−1.77	a	−2.06	a
*AT3G12500*	*PR3*	R	0.06		−0.06	
*AT3G04720*	*PR4*	R	2.43	b	0.35	
*AT5G24770*	*VSP2*	R	0.14		−0.02	
*AT1G32640*	*ATMYC2*	S	−1.70	a	−1.82	a
*AT2G39940*	*COI1*	S	0.25		0.10	

**Table 2:** Expression profiles of selected genes in response to single and combined stresses. Nondeducible gene profiles of selected defense-associated genes and N metabolism are presented. Values represent log2 signal ratios of the fold-changes upon single stresses (N:N limitation; B: *Ea* infection) or combined stresses (N + B:N limitation and *Ea* infection). The column at right indicates the transcriptional response mode according to the categories described by Rasmussen et al. 2013. N:N limitation, B: Bacteria, N + B: Combined stresses (N limitation and bacteria).

Gene Name	Gene Number	N	B	N + B	Response Mode
Response to salicylic acid					
*PR5*	AT1G75040	−2.07	0.88	−0.06	cancelled
*EDS5*	AT4G39030	−1.09	5.67	5.24	prioritized
*PR1*	AT2G14610	−2.71	3.23	2.40	prioritized
ET/JA response and biosynthesis					
*JR1*	AT3G16470	−0.60	−2.01	−0.52	cancelled
*ETR1*	AT1G66340	−0.41	1.14	0.78	cancelled
*LOX3*	AT1G17420	−1.15	2.44	1.96	prioritized
EDS1 pathway					
*FMO1*	AT1G19250	−1.28	5.85	5.02	prioritized
*AtNUDT7*	AT4G12720	−1.10	3.28	2.97	prioritized
WRKY transcription factors					
*WRKY75*	AT5G13080	−1.12	4.20	3.72	prioritized
*WRKY51*	AT5G64810	−1.50	5.93	5.47	prioritized
*WRKY38*	AT5G22570	−1.93	3.68	3.12	prioritized
*WRKY25*	AT2G30250	−1.23	2.53	1.96	prioritized
*WRKY54*	AT2G40750	−1.04	2.22	1.88	prioritized
*WRKY70*	AT3G56400	−1.30	1.69	1.46	prioritized
*WRKY53*	AT4G23810	−1.42	2.24	1.22	prioritized
*WRKY60*	AT2G25000	−1.15	1.77	1.34	prioritized
*WRKY50*	AT5G26170	−1.02	3.45	2.64	prioritized
*WRKY30*	AT5G24110	−1.15	6.11	4.90	prioritized
*WRKY33*	AT2G38470	−1.43	4.63	3.79	prioritized
Resistance genes					
“LRR family protein”	AT5G45510	−1.24	1.87	1.43	prioritized
N metabolism					
*GLN1.3*	AT3G17820	−0.38	−1.02	−0.90	cancelled
*GDH3*	AT3G03910	−0.06	1.21	0.24	cancelled
*AMT1.1*	AT4G13510	−1.13	2.40	1.98	prioritized
*WR3*	AT5G50200	−1.28	3.38	2.63	prioritized
*LHT1*	AT5G40780	−1.30	3.04	2.72	prioritized

## Supplementary Materials

Supplementary materials can be found at http://www.mdpi.com/1422-0067/19/11/3364/s1. Table S1. Genes with the most contrasted response to *E. amylovora* in low and high N. Table S2. Modulation of WRKY TFs by *E.* amylovora in low and high N. Table S3. Response to single and combined stresses of selected N-related genes showing an independent profile. Table S4. Sequence of the gene-specific primers used in this analysis. Figure S1. Nitrate content in Arabidopsis rosette leaves grown under low (0.5 mM) or high (5 mM) nitrate [45]. Figure S2. Modulation profile of previously identified Arabidopsis E. amylovora-responsive genes [21]. Figure S3. Correlation of transcriptome data.

## Abbreviations

B	Bacteria
ET	Ethylene
GO	Gene Ontology
HPI	Hours post inoculation
JA	Jasmonic acid
MYB	MYeloBlastosis
N	Nitrogen
SA	Salicylic acid
TF	Transcription factor

## References

[1] Rasmussen S., Barah P., Suarez-Rodriguez M.C., Bressendorff S., Friis P., Costantino P., Bones A.M., Nielsen H.B., Mundy J. (2013). Transcriptome Responses to Combinations of Stresses in Arabidopsis. Plant Physiol..

[2] Chew Y.H., Halliday K.J. (2011). A stress-free walk from Arabidopsis to crops. Curr. Opin. Biotechnol..

[3] Suzuki N., Rivero R.M., Shulaev V., Blumwald E., Mittler R. (2014). Abiotic and biotic stress combinations. New Phytol..

[4] Nakashima K., Ito Y., Yamaguchi-Shinozaki K. (2009). Transcriptional Regulatory Networks in Response to Abiotic Stresses in Arabidopsis and Grasses. Plant Physiol..

[5] Atkinson N.J., Lilley C.J., Urwin P.E. (2013). Identification of Genes Involved in the Response of Arabidopsis to Simultaneous Biotic and Abiotic Stresses. Plant Physiol..

[6] Dordas C. (2008). Role of nutrients in controlling plant diseases in sustainable agriculture. A review. Agron. Sustain. Dev..

[7] Masclaux-Daubresse C., Daniel-Vedele F., Dechorgnat J., Chardon F., Gaufichon L., Suzuki A. (2010). Nitrogen uptake, assimilation and remobilization in plants: Challenges for sustainable and productive agriculture. Ann. Bot..

[8] Ballini E., Nguyen T.T., Morel J.-B. (2013). Diversity and genetics of nitrogen-induced susceptibility to the blast fungus in rice and wheat. Rice.

[9] Fagard M., Launay A., Clément G., Courtial J., Dellagi A., Farjad M., Krapp A., Soulié M.C., Masclaux-Daubresse C. (2014). Nitrogen metabolism meets phytopathology. J. Exp. Bot..

[10] Daugaard H., Sørensen L., Løschenkohl B. (2016). Effect of Plant Spacing, Nitrogen Fertilisation, Post-Harvest Defoliation and Finger Harrowing in the Control of *Botrytis cinerea* Pers. in Strawberry. Eur. J. Hortic. Sci..

[11] Vega A., Canessa P., Hoppe G., Retamal I., Moyano T.C., Canales J., Gutiérrez R.A., Rubilar J. (2015). Transcriptome analysis reveals regulatory networks underlying differential susceptibility to *Botrytis cinerea* in response to nitrogen availability in *Solanum lycopersicum*. Front. Plant Sci..

[12] Mur L.A.J., Simpson C., Kumari A., Gupta A.K., Gupta K.J. (2017). Moving nitrogen to the centre of plant defence against pathogens. Ann. Bot..

[13] Ward J.L., Forcat S., Beckmann M., Bennett M., Miller S.J., Baker J.M., Hawkins N.D., Vermeer C.P., Lu C., Lin W. (2010). The metabolic transition during disease following infection of *Arabidopsis thaliana* by *Pseudomonas syringae* pv. tomato. Plant. J..

[14] Snoeijers S., Pérez-García A., Joosten M., De Wit P. (2000). The effect of nitrogen on disease development and gene expression in bacterial and fungal plant pathogens. Eur. J. Plant. Pathol..

[15] Dietrich C.R., Ploß K., Heil K. (2004). Constitutive and induced resistance to pathogens in *Arabidopsis thaliana* depends on nitrogen supply. Plant. Cell Environ..

[16] Gupta K.J., Brotman Y., Segu S., Zeier T., Zeier J., Persijn S.T., Cristescu S.M., Harren F.J., Bauwe H., Fernie A.R. (2012). The form of nitrogen nutrition affects resistance against *Pseudomonas syringae* pv. *phaseolicola* in tobacco. J. Exp. Bot..

[17] Tiburcio A.F., Altabella T., Bitrián M., Alcázar R. (2014). The roles of polyamines during the lifespan of plants: From development to stress. Planta.

[18] Huot B., Yao J., Montgomery B.L., He S.Y. (2014). Growth–Defense Tradeoffs in Plants: A Balancing Act to Optimize Fitness. Mol. Plant..

[19] Peng M., Hudson D., Schofield A., Tsao R., Yang R., Gu H., Bi Y.M., Rothstein S.J. (2008). Adaptation of Arabidopsis to nitrogen limitation involves induction of anthocyanin synthesis which is controlled by the NLA gene. J. Exp. Bot..

[20] Lothier J., Gaufichon L., Sormani R., Lemaître T., Azzopardi M., Morin H., Chardon F., Reisdorf-Cren M., Avice J.C., Masclaux-Daubresse C. (2011). The cytosolic glutamine synthetase GLN1;2 plays a role in the control of plant growth and ammonium homeostasis in Arabidopsis rosettes when nitrate supply is not limiting. J. Exp. Bot..

[21] Moreau M., Degrave A., Vedel R., Bitton F., Patrit O., Renou J.P., Barny M.A., Fagard M. (2012). EDS1 contributes to nonhost resistance of *Arabidopsis thaliana* against *Erwinia amylovora*. Mol. Plant-Microbe Interact..

[22] Ruepp A. (2004). The FunCat, a functional annotation scheme for systematic classification of proteins from whole genomes. Nucleic Acids Res..

[23] Alves M., Dadalto S., Gonçalves A., de Souza G., Barros V., Fietto L. (2014). Transcription Factor Functional Protein-Protein Interactions in Plant Defense Responses. Proteomes.

[24] Zhang X., Gou M., Liu C.J. (2014). Arabidopsis Kelch Repeat F-Box Proteins Regulate Phenylpropanoid Biosynthesis via Controlling the Turnover of Phenylalanine Ammonia-Lyase. Plant Cell..

[25] Yang Y., He M., Zhu Z., Li S., Xu Y., Zhang C., Singer S.D., Wang Y. (2012). Identification of the dehydrin gene family from grapevine species and analysis of their responsiveness to various forms of abiotic and biotic stress. BMC Plant Biol..

[26] Galon Y., Nave R., Boyce J.M., Nachmias D., Knight M.R., Fromm H. (2008). Calmodulin-binding transcription activator (CAMTA) 3 mediates biotic defense responses in Arabidopsis. FEBS Lett..

[27] Birkenbihl R.P., Liu S., Somssich I.E. (2017). Transcriptional events defining plant immune responses. Curr. Opin. Plant Biol..

[28] Camanes G., Pastor V., Cerezo M., García-Andrade J., Vicedo B., García-Agustín P., Flors V. (2012). A deletion in NRT2.1 attenuates *Pseudomonas syringae*-induced hormonal perturbation, resulting in primed plant defenses. Plant Physiol..

[29] Dechorgnat J., Patrit O., Krapp A., Fagard M., Daniel-Vedele F. (2012). Characterization of the Nrt2.6 Gene in Arabidopsis thaliana: A Link with Plant Response to Biotic and Abiotic Stress. PLoS ONE.

[30] Liu G., Ji Y., Bhuiyan N.H., Pilot G., Selvaraj G., Zou J., Wei Y. (2010). Amino Acid Homeostasis Modulates Salicylic Acid-Associated Redox Status and Defense Responses in Arabidopsis. Plant Cell.

[31] Pastor V., Gamir J., Camañes G., Cerezo M., Sanchez-Bel P., Flors V. (2014). Disruption of the ammonium transporter AMT1.1 alters basal defenses generating resistance against *Pseudomonas syringae* and *Plectosphaerella cucumerina*. Front. Plant Sci..

[32] Titarenko E., Rojo E., Leon J., Sanchez-Serrano J.J. (1997). Jasmonic acid-dependent and -independent signaling pathways control wound-induced gene activation in *Arabidopsis thaliana*. Plant Physiol..

[33] Walters D.R., Bingham I.J. (2007). Influence of nutrition on disease development caused by fungal pathogens: Implications for plant disease control. Ann. Appl. Biol..

[34] Massad T.J., Dyer L.A., Vega C.G. (2012). Costs of defense and a test of the carbon-nutrient balance and growth-differentiation balance hypotheses for two co-occurring classes of plant defense. PLoS ONE.

[35] Coolen S., Proietti S., Hickman R., Davila Olivas N.H., Huang P.P., Van Verk M.C., Van Pelt J.A., Wittenberg A.H., De Vos M., Prins M. (2016). Transcriptome dynamics of Arabidopsis during sequential biotic and abiotic stresses. Plant. J..

[36] Atkinson N.J., Urwin P.E. (2012). The interaction of plant biotic and abiotic stresses: From genes to the field. J. Exp. Bot..

[37] Degrave A., Fagard M., Perino C., Brisset M.N., Gaubert S., Laroche S., Patrit O., Barny M.A. (2008). *Erwinia amylovora* type three–secreted proteins trigger cell death and defense responses in *Arabidopsis thaliana*. Mol. Plant Microb. Interact..

[38] Venisse J.-S., Malnoy M., Faize M., Paulin J.-P., Brisset M.-N. (2002). Modulation of defense responses of *Malus* spp. during compatible and incompatible interactions with *Erwinia amylovora*. Mol. Plant-Microbe Interact..

[39] De Bernonville T.D., Gaucher M., Flors V., Gaillard S., Paulin J.P., Dat J.F., Brisset M.N. (2012). T3SS-dependent differential modulations of the jasmonic acid pathway in susceptible and resistant genotypes of *Malus* spp. challenged with *Erwinia amylovora*. Plant Sci..

[40] Eulgem T. (2005). Regulation of the Arabidopsis defense transcriptome. Trends Plant Sci..

[41] Tao Y., Xie Z., Chen W., Glazebrook J., Chang H.S., Han B., Zhu T., Zou G., Katagiri F. (2003). Quantitative Nature of Arabidopsis Responses during Compatible and Incompatible Interactions with the Bacterial Pathogen *Pseudomonas syringae*. Plant Cell.

[42] Larionov A., Krause A., Miller W. (2005). A standard curve based method for relative real time PCR data processing. BMC Bioinform..

[43] Ceccato L., Masiero S., Roy D.S., Bencivenga S., Roig-Villanova I., Ditengou F.A., Palme K., Simon R., Colombo L. (2013). Maternal control of PIN1 is required for female gametophyte development in Arabidopsis. PLoS ONE.

[44] Storey J.D., Tibshirani R. (2003). Statistical significance for genomewide studies. Proc. Natl. Acad. Sci. USA.

[45] Miranda K.M., Espey M.G., Wink D.A. (2001). A Rapid, Simple Spectrophotometric Method for Simultaneous Detection of Nitrate and Nitrite. Nitric Oxide-Biol. Chem..

