# Supplementary material for: Nitrogen Limitation Alters the Response of Specific Genes to Biotic Stress
Source: Int J Mol Sci. 2018 Oct 27;19(11):3364. doi: 10.3390/ijms19113364 (PMC6275003; doi:10.3390/ijms19113364)
Supplement: Supplementary file 1 [file ijms-19-03364-s001.pdf]

Table S1. Genes with the most contrasted response to *Ea* in low and full N. Values are log2 signal ratios between *Ea*-infected and mock plants at low (0.5 mM NO_3^-) and full N (5 mM NO_3^-). Top: genes repressed by *Ea* only in plants grown in low N; middle: genes induced by *Ea* only in low N or more induced in low N; bottom: genes repressed by *Ea* only in full N or more repressed in full N. Asterisk (*) indicate a significant difference between mock and infected plants according to the Bonferroni test (*P*-value < 0.05).

AGI	Name	0.5 mM NO_3^- Ea vs mock		5 mM NO_3^- Ea vs mock	
AT1G09350	Galactinol synthase 3	-3.37	*	-1.42	
AT3G19350	Polyadenylate-binding protein-related	-3.31	*	-0.85	
AT2G12905	Unknown protein	-2.07	*	0.08	
AT2G01021	Unknown protein	-2.01	*	-0.09	
AT1G26630	Eukaryotic elongation factor 5A-2	-1.79	*	0.06	
AT1G47450	Unknown protein	-1.78	*	0.17	
AT2G07681	Cytochrome C assembly protein	-1.72	*	0.28	
AT2G07681	Cytochrome C assembly protein	-1.62	*	0.29	
AT4G05631	Unknown protein	-1.59	*	0.51	
AT2G07681	Cytochrome C assembly protein	-1.52	*	0.39	
AT1G61120	Terpene synthase	5.40	*	3.47	*
AT1G15670	Kelch repeat-containing F-box family protein	5.39	*	3.23	*
AT3G16530	Legume lectin family protein	4.61	*	2.59	*
AT5G35525	Plac8 family protein	3.92	*	1.97	*
AT1G80440	Kelch repeat-containing F-box family protein	3.61	*	-0.07	
AT3G46660	UDP-Glycosyltransferase	3.10	*	1.11	
AT5G54610	ANKYRIN (ANK)	2.83	*	0.59	
AT1G26800	Zinc finger family protein	2.74	*	0.20	
AT3G04720	Pathogenesis-related4 (PR4)	2.43	*	0.35	
AT2G14560	Unknown protein	2.02	*	-0.44	
AT3G49940	LOB domain-containing protein 38	1.23	*	-0.71	
AT3G48360	BTB and TAZ domain protein 2	-1.84	*	-4.16	*
AT5G19190	Unknown protein	-1.20		-3.23	*
AT4G32480	Unknown protein	-0.96		-3.23	*
AT5G66580	Unknown protein	-0.65		-2.61	*
AT1G49130	Zinc finger family protein	0.10		-2.17	*
AT3G16470	Jacalin lectin family protein (JR1)	0.08		-2.01	*
AT1G23390	Kelch repeat-containing F-box family protein	0.24		-1.97	*
AT5G03350	Legume lectin family protein	0.91		-1.88	*
AT3G57240	β-1.3-glucanase (BG3)	1.06		-1.63	*

Table S2. Modulation of *WRKY* TFs by *Ea* in low and full N. Values are log2 signal ratios between infected and mock plants grown 0.5 and 5 mM NO_3^-. Two levels of significance threshold are presented according to the Bonferroni statistical test (a: *P*-value < 0.05; b: *P*-value < $1*10^{-8}$).

AGI	Name	0.5 mM NO_3^-		5 mM NO_3^-	
		Ea vs mock		Ea vs mock	
AT3G58710	"WRKY69 (WRKY DNA-binding protein 69); transcription factor"	-1.21		-1.26	a
AT4G30935	"WRKY32 (WRKY DNA-binding protein 32); transcription factor"	-0.79		-0.30	
AT2G03340	"WRKY3 (WRKY DNA-binding protein 3); transcription factor"	-0.58		-1.55	a
AT5G56270	"WRKY2 (WRKY DNA-binding protein 2); transcription factor"	-0.41		-0.01	
AT4G26640	"WRKY20 (WRKY DNA-binding protein 20); transcription factor"	-0.28		-0.31	
AT4G26640	"WRKY20 (WRKY DNA-binding protein 20); transcription factor"	-0.15		-0.04	
AT2G47260	"WRKY23 (WRKY DNA-binding protein 23); transcription factor"	-0.15		-0.22	
AT5G41570	"WRKY24 (WRKY DNA-binding protein 24); transcription factor"	-0.14		-0.04	
AT1G64000	"WRKY56 (WRKY DNA-binding protein 56); transcription factor"	-0.13		0.11	
AT5G28650	"WRKY74 (WRKY DNA-binding protein 74); transcription factor"	-0.09		-0.05	
AT2G46130	"WRKY43 (WRKY DNA-binding protein 43); transcription factor"	-0.01		0.09	
AT2G34830	"WRKY35 (WRKY DNA-binding protein 35.); transcription factor"	0.00		-0.06	
AT4G26440	"WRKY34 (MICROSPORE-SPECIFIC PROMOTER 3); transcription factor"	0.00		-0.07	
AT1G29280	"WRKY65 (WRKY DNA-binding protein 65); transcription factor"	0.04		-0.51	
AT1G30650	"WRKY14 (WRKY DNA-binding protein 14); transcription factor"	0.04		0.11	
AT1G69310	"WRKY57 (WRKY DNA-binding protein 57); transcription factor"	0.05		-0.28	
AT1G55600	"WRKY10 (MINISEED3); transcription factor"	0.05		0.17	
AT3G62340	"WRKY68 (WRKY DNA-binding protein 68); transcription factor"	0.19		0.03	
AT1G66600	"WRKY63 (WRKY DNA-binding protein 63); transcription factor"	0.24		0.14	
AT3G01080	"WRKY58 (WRKY DNA-binding protein 58); transcription factor"	0.32		0.10	
AT4G39410	"WRKY13 (WRKY DNA-binding protein 13); transcription factor"	0.46		0.25	
AT4G12020	"WRKY19 (WRKY DNA-binding protein 19); transcription factor"	0.47		0.29	
AT1G66550	"WRKY67 (WRKY DNA-binding protein 67); transcription factor"	0.63		1.33	a
AT1G66560	"WRKY64 (WRKY DNA-binding protein 64); transcription factor"	0.69		1.57	a
AT2G30590	"WRKY21 (WRKY DNA-binding protein 21); transcription factor"	0.92		0.51	
AT1G13960	"WRKY4 (WRKY DNA-binding protein 4); transcription factor"	0.94		0.75	
AT4G01720	"WRKY47 (WRKY DNA-binding protein 47); transcription factor"	1.09		1.49	a
AT4G04450	"WRKY42 (WRKY DNA-binding protein 42); transcription factor"	1.32		1.46	a
AT1G69810	"WRKY36 (WRKY DNA-binding protein 36); transcription factor"	1.65	a	1.73	a
AT5G52830	"WRKY27 (WRKY DNA-binding protein 27); transcription factor"	1.70	a	1.74	a
AT5G43290	"WRKY49 (WRKY DNA-binding protein 49); transcription factor"	1.80	a	2.49	b
AT4G31550	"WRKY11 (WRKY DNA-binding protein 11); transcription factor"	1.97	a	2.42	b
AT5G07100	"WRKY26 (WRKY DNA-binding protein 26); transcription factor"	1.99	a	2.51	b
AT1G80590	"WRKY66 (WRKY DNA-binding protein 66); transcription factor"	2.00	a	2.32	b
AT1G18860	"WRKY61 (WRKY DNA-binding protein 61); transcription factor"	2.31	b	2.81	b
AT5G01900	"WRKY62 (WRKY DNA-binding protein 62); transcription factor"	2.34	b	2.73	b
AT1G29860	"WRKY71 (WRKY DNA-binding protein 71); transcription factor"	2.37	b	2.34	b
AT2G21900	"WRKY59 (WRKY DNA-binding protein 59); transcription factor"	2.41	b	2.98	b
AT2G25000	"WRKY60 (WRKY DNA-binding protein 60); transcription factor"	2.49	b	1.77	a
AT2G24570	"WRKY17 (WRKY DNA-binding protein 17); transcription factor"	2.55	b	2.43	b
AT4G23810	"WRKY53 (WRKY DNA-binding protein 53); transcription factor"	2.63	b	2.24	b
AT4G24240	"WRKY7 (WRKY DNA-binding protein 7); transcription factor"	2.66	b	2.63	b
AT3G56400	"WRKY70 (WRKY DNA-binding protein 70); transcription factor"	2.76	b	1.69	a
AT3G04670	"WRKY39 (WRKY DNA-binding protein 39); transcription factor"	2.90	b	2.83	b
AT2G40750	"WRKY54 (WRKY DNA-binding protein 54); transcription factor"	2.92	b	2.22	b
AT2G23320	"WRKY15 (WRKY DNA-binding protein 15); transcription factor"	3.14	b	2.93	b
AT2G30250	"WRKY25 (WRKY DNA-binding protein 25); transcription factor"	3.18	b	2.53	b
AT5G46350	"WRKY8 (WRKY DNA-binding protein 8); transcription factor"	3.38	b	3.94	b
AT3G01970	"WRKY45 (WRKY DNA-binding protein 45); transcription factor"	3.51	b	3.45	b
AT4G11070	"WRKY41 (WRKY DNA-binding protein 41); transcription factor"	3.54	b	4.08	b
AT4G31800	"WRKY18 (WRKY DNA-binding protein 18); transcription factor"	3.65	b	4.09	b
AT5G26170	"WRKY50 (WRKY DNA-binding protein 50); transcription factor"	3.66	b	3.45	b
AT4G18170	"WRKY28 (WRKY DNA-binding protein 28); transcription factor"	3.71	b	3.00	b
AT5G49520	"WRKY48 (WRKY DNA-binding protein 48); transcription factor"	3.78	b	4.25	b
AT2G40740	"WRKY55 (WRKY DNA-binding protein 55)"	3.80	b	4.61	b
AT1G62300	"WRKY6 (WRKY DNA-binding protein 6); transcription factor"	3.85	b	3.03	b
AT1G68150	"WRKY9 (WRKY DNA-binding protein 9); transcription factor"	3.92	b	4.39	b
AT5G15130	"WRKY72 (WRKY DNA-binding protein 72); transcription factor"	3.94	b	4.11	b
AT4G22070	"WRKY31 (WRKY DNA-binding protein 31); transcription factor"	4.39	b	4.76	b
AT5G13080	"WRKY75 (WRKY DNA-binding protein 75); transcription factor"	4.83	b	4.20	b
AT5G22570	"WRKY38 (WRKY DNA-binding protein 38); transcription factor"	5.05	b	3.68	b
AT2G38470	"WRKY33 (WRKY DNA-binding protein 33); transcription factor"	5.22	b	4.63	b
AT4G01250	"WRKY22 (WRKY DNA-binding protein 22); transcription factor"	5.43	b	5.75	b
AT1G80840	"WRKY40 (WRKY DNA-binding protein 40); transcription factor"	5.59	b	5.29	b
AT4G23550	"WRKY29 (WRKY DNA-binding protein 29); transcription factor"	5.62	b	6.34	b
AT2G46400	"WRKY46 (WRKY DNA-binding protein 46); transcription factor"	5.65	b	5.38	b
AT5G24110	"WRKY30 (WRKY DNA-binding protein 30); transcription factor"	6.06	b	6.11	b
AT5G64810	"WRKY51 (WRKY DNA-binding protein 51); transcription factor"	6.97	b	5.93	b

Table S3. Response to single and combined stresses of selected N-related genes showing an independent profile. Values represent log 2 signal ratios of the fold-changes upon single stresses (N: N limitation; B: *Ea* infection) or combined stresses (N+B: N limitation and *Ea* infection). Most N-related genes that were modulated by at least one of the single stresses presented an independent profile in response to combined stresses (presented in this table). A few N-related genes presented non-predictable profiles in response to combined stresses and are presented in Table 2. N: N limitation; B: *Ea* infection; N+B: N limitation and *Ea* infection.

Name	AGI	N	B	N+B	Response mode
AMT1.2	AT1G64780	0.17	-4.09	-4.18	independent
AAP3	AT1G77380	-0.46	3.33	3.47	independent
AMT2.1	AT2G38290	-0.73	3.55	2.91	independent
ASPGB1	AT3G16150	-0.09	1.55	1.59	independent
ASPGB1	AT3G16150	-0.09	1.55	1.59	independent
CARB	AT1G29900	-0.61	-1.15	-1.55	independent
GABA-T	AT3G22200	0.28	1.11	1.15	independent
GAD1	AT5G17330	-0.05	6.84	6.34	independent
GAD2	AT1G65960	-0.57	-1.46	-1.92	independent
GAT1	AT2G15570	-0.51	1.93	1.50	independent
GDH2	AT5G07440	-0.62	1.96	1.98	independent
GLN1.1	AT5G37600	-0.62	1.96	1.72	independent
GLN1.2	AT1G66200	-0.51	-2.42	-2.04	independent
GLN1.4	AT5G16570	0.29	3.43	3.57	independent
GLU1	AT5G04140	0.05	-2.23	-2.11	independent
GS2	AT5G35630	-0.48	-1.61	-2.51	independent
NIA1	AT1G77760	-2.37	-0.89	-1.60	independent
Nitr	AT1G68570	-0.03	-4.27	-4.08	independent
NRT1.1	AT1G12110	-0.69	-1.78	-1.92	independent
NRT1.5	AT1G32450	0.16	-1.58	-1.66	independent
NRT1.7	AT1G69870	-0.17	-1.67	-2.07	independent
NRT2.1	AT1G08090	0.40	2.34	1.64	independent
NRT2.5	AT1G12940	0.09	4.53	4.14	independent
NRT2.6	AT3G45060	-0.09	6.23	6.06	independent
NRT2.7	AT5G14570	-0.20	-2.62	-2.69	independent

Table S4. Sequence of the gene-specific primers used in this analysis.

A. thaliana genes	LP	RP
PR1	TCTTCCCTCGAAAGCTCAAGA	GTGCCTGGTTGTGAACCCTTA
PR5	GCCGTGGAGCTAACGATAAG	GCGTAGCTATAGGCGTCAGG
WRKY33	CATTTCTGCCACCAAAGGAT	TTGATTGGTTTTATTCCGTGTTC
WRKY60	GGGTCACCCCCATTTTATCT	GGCATCACATTCTTCTCTAATGG

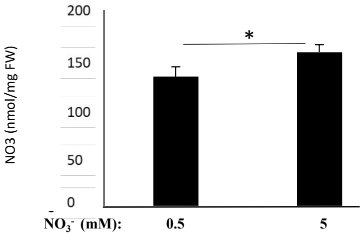

Figure S1. Nitrate content in Arabidopsis rosette leaves grown under low (0.5 mM) or full (5 mM) nitrate. Nitrate content was measured using the Miranda method [45] in 25 mg ground and frozen leaves. Each biological replicate corresponds to 4 leaves of a single plant. Values presented correspond to the mean and SE of three individual replicates. The asterisk (*) indicates a significant difference between the two N nutritional conditions according to the Mann-Whitney test (*P*-value < 0.05).

		This study		
		HN	LN	Moreau
**Response to salicylic acid**				
AT3G52430	PAD4	4,51	5,15	3,23
AT1G74710	ICS1	5,73	6,14	3,48
AT4G39030	EDS5	6,03	6,13	2,22
AT3G48090	EDS1	3,82	3,85	1,94
AT1G21270	WAK2	1,41	1,63	1,46
AT1G64280	NPR1	1,66	2,14	1,03
**Response to jasmonic acid and ethylene**				
AT4G17500	ERF1	1,92	1,93	
AT3G12500	CHI-B			
AT1G66340	ETR1			
AT1G75830	PDF1.1	1,22	1,86	-1,25
AT5G42650	AOS	-2,13		-1,28
AT3G16470	JL	-2,01		-0,95
AT1G13280	AOX	-2,61		-0,95
AT1G19640	JMT			
AT2G43710	SSI2	-1,36		
AT2G06050	OPR3			
AT2G39940	COI1			
**Response to oxidative stress**				
AT3G22370	AOX1A	2,72	2,63	2,01
AT1G64060	RbohF	1,53	0,91	1,69
**Flavonoid biosynthesis**				
AT5G13930	CHS	-2,00	-1,77	
AT5G08640	FLS	-3,83		-1,34
**Resistance genes**				
AT1G66090	NBS-LRR	5,11	5,30	4,63
AT3G14470	NBS-LRR	3,77	3,88	2,02
AT4G11170	NBS-LRR	7,45	7,00	1,54
AT5G51630	NBS-LRR	3,43	3,27	0,96
AT4G14370	NBS-LRR	2,96	2,68	1,39
AT4G33300	NBS-LRR	2,66	2,80	1,92
AT5G45510	NBS-LRR	1,87	2,66	1,17
AT1G17615	NBS-LRR	1,58	1,82	
AT5G38350	NBS-LRR	1,29	0,93	
AT4G19530	NBS-LRR	-1,75		-1,25

Figure S2. Modulation profile of previously identified Arabidopsis *Ea*-responsive genes. In the three columns, the values represent the log2 ratios between *Ea*-infected and mock-treated five-week-old *A. thaliana* Col-0 leaves. The first two columns correspond to data from the present study for plants grown in full (5 mM; HN) or low NO_3^- (0.5 mM; LN). The last column on the right (Moreau) corresponds to a previous dataset corresponding to plants grown in standard growth conditions [21]. Values corresponding to *Ea*-induced and repressed genes are highlighted respectively in pink and green. The log2 ratio for genes not significantly modulated according to the Bonferroni statistical test (*P*-value < 0.05) appear in black.

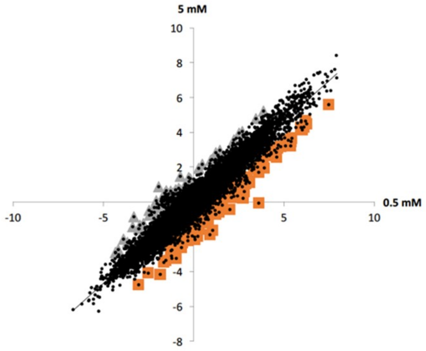

Figure S3. Correlation of transcriptome data. Correlation between log2 values of the fold-changes upon *Ea* infection in plants grown in low (0.5 mM NO_3^-) and full N (5 mM NO_3^-). Each black dot represents a single gene. Gray and orange dots correspond to genes with the most contrasted response to *Ea* in low and full N; Grey: higher modulation by *Ea* in 5 mM NO_3^-; orange: higher modulation by *Ea* in 0.5 mM NO_3^-.
